# Supplementary material for: Aβ-driven nuclear pore complex dysfunction alters activation of necroptosis proteins in a mouse model of Alzheimer’s disease
Source: eLife. 2025 Mar 25;13:RP92069. doi: 10.7554/eLife.92069 (PMC11936419; doi:10.7554/eLife.92069)
Supplement: Figure 2—source data 1. [file elife-92069-fig2-data1.zip › Figure 2 Source Data 1/Figure 2 - Source Data File 1.pdf]

Full length Western blots from Figure 2H

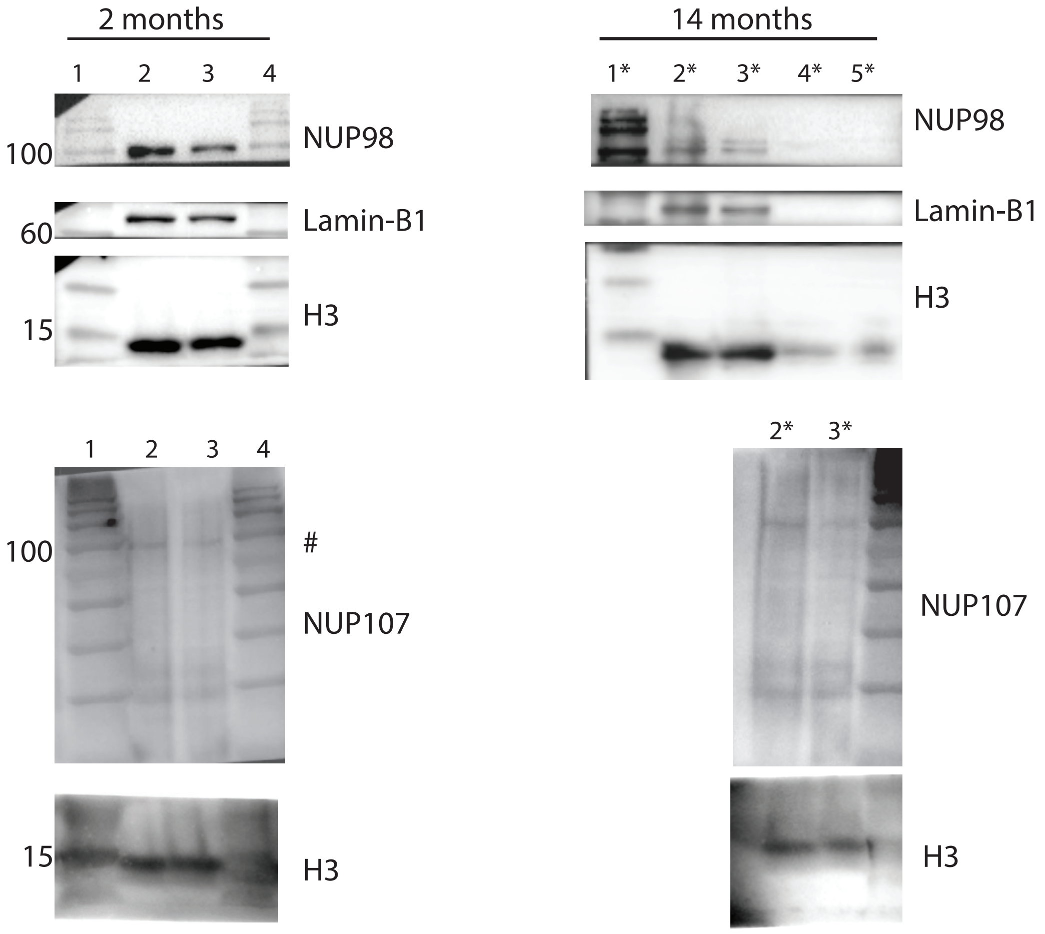

Due to limited input material for brain-derived nuclei, we often have to cut full length blots into strips and incubate them with appropriate antibodies. The blots above are the full strips we used to probe for the protein.

Blots were immunoassayed for antibodies against NUP98, Lamin-B1, NUP107, and Histone H3. Lanes 1, 4, and 1* are protein ladder, lanes 2 and 2* are WT nuclear lysates, lane 3 and 3* are *App* KI nuclear lysates, lane 4* is WT total input lysate, and lane 5* is *App* KI nuclear input lysate. Quantification of blots is described in the methods and materials section.
